# Factors Associated with HIV Testing among Reproductive Women Aged 15–49 Years in the Gambia: Analysis of the 2019–2020 Gambian Demographic and Health Survey

**DOI:** 10.3390/ijerph19084860

**Published:** 2022-04-16

**Authors:** Michael Deynu, Kingsley Agyemang, Nana Anokye

**Affiliations:** Division of Global Public Health, Department of Health Sciences, College of Health, Medicine and Life Sciences, Brunel University London, London UB8 3PH, UK; michael.deynu@yahoo.com (M.D.); kingsley.agyemang@brunel.ac.uk (K.A.)

**Keywords:** reproductive women, human immunodeficiency virus testing

## Abstract

Voluntary counselling and testing for Human Immunodeficiency Virus (HIV) and Acquired Immune Deficiency Syndrome (AIDS) has always been one of the key policy interventions in the management and control of HIV/AIDS transmission. However, the prevalence of HIV testing among reproductive women in the Gambia remains low despite near universal information about HIV and Sexually Transmitted Infections (STIs) in the Gambia. Understanding factors influencing HIV testing uptake provides empirical data for the development of targeted evidenced-based strategies aimed at enhancing HIV testing uptake. Therefore, this study examined the factors associated with HIV testing among reproductive women aged 15–49 years in the Gambia. Data on weighted sample of 11,865 women from the 2019–2020 Gambia Demographic and Health Survey were analyzed in this study. Chi square, bivariate and multivariate logistic regression models were fitted and analysis conducted through Complex Samples Analysis in Statistical Package for Social Sciences (SPSS). Level of significance was set at *p* < 0.05 and 95% CI. Further analysis was conducted to determine the variability in HIV testing among women stratified by rural and urban centers. Prevalence of HIV testing among reproductive women was 42.1% (95% CI = 40.1–44.2%) in the Gambia. Women aged 20–24 years and 25–29 years (aOR = 3.10, 95% CI = 2.51–3.83) and (aOR = 4.52, 95% CI = 3.61–5.54) were more likely to test for HIV than those aged 15–19 years, respectively. Married women (aOR = 5.90, 95% CI = 4.84–7.02) were more likely to test for HIV compared to those who were not in any union. Respondents with higher education in urban centers (aOR = 2.65, 95% CI = 2.08–3.86) were likely to test for HIV compared to those in rural areas. HIV testing in the Gambia among reproductive women is low. Age, marital status, wealth index, place of residence, educational level, recent sexual activity, previous history of risky sexual behaviors, and history of an STI were associated with HIV testing. Health interventions targeted at increasing HIV testing uptake should factor in these.

## 1. Introduction

The global burden of Human Immunodeficiency Virus/Acquired Immune Deficiency Syndrome (HIV/AIDS) currently stands at about 38 million with an approximate 36.2 million being adults and 1.8 million children [1]. The burden of new HIV infections declined from 3.4 million infections in 2011 to 2.1 million in 2013 globally [2]. Notwithstanding this feat, HIV/AIDS and its transmission still poses a significant public health threat to the global population [3], with sub-Saharan African countries reporting two-thirds of all total new infections [4]. The HIV prevalence in the Gambia stood at 1.65% for HIV-1 and 0.07% for HIV-2 as at 2017 [5,6] and 1.8% in 2020 [7]. Globally, 81% of persons living with HIV are aware of their status and nearly 19 million people do not know of their sero-status, even though this figure has dropped to about 7.1 million in 2019 [2]. Human Immunodeficiency Virus (HIV) testing and counselling is one major public health intervention that involves the counselling, testing and treatment with the view to reducing the transmission of HIV/AIDS as well as the associated health burden [2].

The relative importance of Voluntary Counselling and Testing (VCT) sessions cannot be underestimated. Voluntary HIV counselling and testing has long been one of the key policy strategies in managing HIV transmission [8].

This is because individuals who attend these sessions become informed of their status and gain relevant knowledge on how to avoid risky health behaviors in order to protect themselves as well as others [8]. These sessions also serve as avenues through which individuals can access HIV treatment and other interventions including social and emotional support, thereby enabling persons living with HIV/AIDS to cope with the anxiety and apprehension associated with the disease and its related burden [8]. The United Nations (UN), to further promote testing and treatment for persons living with HIV/AIDS, declared and endorsed the agenda 90-90-90 with the aim that by 2020, 90% of all persons living with HIV will know their status, 90% of all people with HIV diagnosis will receive Anti-Retroviral Treatment (ARTs), and 90% of all people receiving ARTs will achieve viral suppression [9,10,11]. This is relevant within the context of ending the HIV pandemic by 2030. It was envisaged that when this three-pronged approach is achieved, about 73% of all people living with HIV globally will be virally suppressed. Achieving these targets by 2020 would have enabled the global community to end the HIV epidemic by 2030, thereby increasing the health status of peoples as well its economic benefits. To achieve these targets, countries may have to review current programs with the view to identifying the potential factors or barriers that may hinder the realization of these goals. Gambia in recent times has made some progress, for instance, making HIV testing among pregnant women mandatory during antenatal visits. However, more needs to be done.

Despite these interventions and the high prevalence and risks of acquiring HIV among adult women in Sub-Saharan Africa, women’s access to and utilization of HIV counselling and testing services remains low [3,12,13] with adolescent girls and reproductive women disproportionately accounting for over 60% of new HIV infections occurring each day globally [14].

In the Gambia, HIV testing among young women has seen a steady decline from 22% in 2013 to 19% in 2019–2020 resulting from the apparent lack in accessing health services as well as the presence of other socio-demographic barriers that hinders access to healthcare [15]. Additionally, HIV testing coverage among women in the Gambia is, however, at 39% with about 28% of them having reported condom usage during their last sexual intercourse and 27% of them having comprehensive knowledge about HIV [15]. General awareness about HIV/AIDS is high among women [3], however this does not translate into increased uptake of HIV testing practices [3].

This reveals a sharp disconnect between the generally high awareness rate and the low uptake of HIV testing that needs to be investigated empirically. Other studies conducted in Ethiopia and other parts of Eastern Africa have identified age, gender, marital status, educational status, socioeconomic status, area of residence, and wealth index [4,7,9,16] as independent predictors of HIV testing among young women. However, in the Gambia, previous studies conducted around HIV have largely focused on HIV knowledge and risk behaviors among men who have sex with men [17], trends of HIV-1 and HIV-2 [18]. Others also focused on the reasons individuals fail to return for HIV test results [19].

Even though findings from these studies are still valid and relevant, population dynamics and other health indicators have changed in recent times. In addition, no current study to the best of our knowledge has so far examined the determinants of HIV Testing in the Gambia using current data at the national level. Again, HIV Testing has been found to vary depending on whether an individual resides in an urban or rural area in other jurisdictions. Evidence from studies conducted in Ethiopia and across the United States of America [20,21,22,23] have reported that women living in rural areas have substantially lower odds of HIV Testing compared to those in urban areas. In the Gambia however, no study has been identified to have been conducted exploring the variabilities in HIV testing across one’s place of residence (rural/urban). As such there are limited data on the rural/urban variations in HIV Testing among reproductive women in the Gambia.

This empirical data are crucial in identifying areas with low HIV Testing rates to aid in the design and implementation of targeted HIV Testing interventions as well as guiding public health officials and policy makers to allocate equitably resources to low testing areas. Therefore, this study aimed to investigate the factors associated with HIV testing among women in the Gambia.

## 2. Materials and Methods

### 2.1. Study Participants and Data Sources

This cross-sectional secondary analysis was carried out on reproductive women aged 15–49 years in the Gambia who have either taken an HIV test or not. Data for this sample were derived from the 2019–2020 Gambian Demographic and Health Survey dataset [24]. To access the data for analysis, we sought permission from DHS of the ICF International that hosts the data. This was done through an application after registering the research project and describing how the data will be used as well as the analysis process. Access was then granted through a formal communication. The DHS surveys are nationally representative surveys that collect data on fertility levels and preferences, contraceptive use, maternal and child health, infant, child, and neonatal mortality levels. It also collects information on maternal mortality, gender, nutrition, awareness about HIV/AIDS, self-reported sexually transmitted infections (STIs), and other health issues relevant to the achievement of the Sustainable Development Goals (SDGs) [25]. The survey participants in these large national surveys were selected representatively from all the eight (8) regions of the Gambia, stratified into rural and urban areas.

Data collection was done through multistage sampling design. The first stage comprised the selection of Enumeration Areas (clusters) from an updated master sampling frame designed in previous surveys. This is usually done using systematic sampling with a probability proportional to the population size and the number of households within the clusters. This is followed by the second stage through listing of households within the selected clusters, providing sampling frame where the households were randomly selected from all the clusters to provide enough estimates for key indicators with acceptable precision. The 2019–2020 Gambian Demographic and Health Survey generated data from 6985 households from which 11,865 reproductive women aged 15–49 years were sampled from November 2019 to March 2020. The detailed methodology of the survey design, sampling, survey tools used and the data collection methods are described here [15].

### 2.2. Study Variables and Measurements

#### 2.2.1. Dependent Variable

The main outcome variable in this secondary analysis was “Ever been tested for HIV/AIDS”. It was measured in the 2019–2020 Gambian Demographic and Health Survey (DHS) as a binary variable with the response categories as 0 = No (for reproductive women who have not tested for HIV/AIDS) and 1 = Yes (for reproductive women who have tested for HIV/AIDS) within the last 12 months prior to the survey. Therefore, this variable as used in the analysis evaluated HIV testing among women aged 15–19 years within the last 12 months.

#### 2.2.2. Independent Variables

Based on the interaction between the constructs of extended Theory of Planned Behavior (TPB) [26] and findings from previous literature [4,7,9,16,17,18,19,20,21,22,23], the following variables grouped into Sociodemographic Variables (Age, Current Marital Status, Type of place of Residence, Highest Educational level, Religion, Ethnicity, Wealth Index combined, Respondent currently working, and Covered by Health Insurance) were the independent variables analyzed in this study. Other independent variables included HIV-Related Knowledge variables (Ever heard of HIV and other STIs, Knowing a place to get HIV test, Knowledge and use of HIV testing kits, Place where last HIV test was taken, Receiving results for test undertaken and seeking treatment for STI infections) and variables that explored risks associated with their sexual behaviors (Risky Sexual Behaviours)-(Had any STI, Relationship with recent partner, Having had sex for gifts/cash in the last 12 months, Had genital sores, Had genital discharges, Recent sexual activities, Recent sexual activity, and Number of sex partner including spouse).

Age was categorized into (15–19, 20–24, 25–29, 30–34, 35–39, 40–44, 45–49), Current marital status (Never in union, Married, Living with partner, Widowed, Divorced, No longer living together/separated), Religion (Islam, Christian and Other), Wealth Index (Low, Middle, High), Ethnicity (Mandinka/Jahanka, Wollof, Jola/Karoninka, Fula/Tukulur/Lorobo, Serere, Sarahule, Creole/Aku Marabout, Manjago, Bambara and Other), Highest Educational level (No education, Primary, Secondary, Higher), Type of place of residence (Urban, Rural), Respondent currently working (No, Yes), and Insurance coverage (No, Yes). Ever heard of HIV and STIs were both coded as binary (No, Yes), Knows a place to get HIV test (No and Others, Yes), Knowledge and use of test kits (Unknown and Known), Place where last HIV test was taken (Government Hospitals, Government health centers/clinic/health posts, Private hospitals/services, and Others). Received results for last HIV test was (No & Others, Yes), Recent Sexual activity (Never had sex, Active in last 4 weeks, and Others), Relationship with most recent sex partner (Spouse, Others), Number of sex partners including spouse in last 12 months (None, One or more), Had sex in the last 12 months in return for gifts/cash (No, Yes and Others), Having genital sores (No, Yes, Don’t Know), Having genital discharges (No, Yes, Don’t Know), Seeking treatment for last STI infection (No, Yes, Others), Had any STI (No, Yes).

To avoid small cell count problems during regression, Religion was recoded into two categories (Islam, Christianity and Others), and Wealth Index into Low, Middle and High.

### 2.3. Data Analysis

Data analysis was conducted through following stages; Data Cleaning and Integrity Checks, Univariate Analysis and analysis of missing data, Bivariate Analysis, Multivariate Regression Analysis (Binary Logistic Regression) and Sub-group Analysis using the IBM Statistical Package for Social Sciences (SPSS) version 26 (IBM, Armonk, NY, USA) with level of significance set at *p* < 0.05 and 95% Confidence Interval (95% CI). The characteristics of the study variables were described using frequencies and percentages because they were categorical in nature. Pearson’s Chi Square statistics was then performed to investigate associations between the dependent variable and the predictors for HIV testing. Binary logistic regression was fitted to identify the influencing factors for HIV testing. The regression model was again fitted with the predictors to empirically investigate how the predictors of HIV testing compares among the rural and urban dwellers in the Gambia.

This additional analysis was to identify factors that inform geographically sensitive public health interventions, more so when other studies have reported variations in HIV testing across geographies [20,21,22,23,27] often resulting in area-specific barriers to HIV testing. This is relevant in the context of policy formulation as it aims to generate empirical findings that could help in the design of appropriate and context specific interventions targeted at increasing the uptake of HIV Testing in the Gambia. In this analysis, the independent variable “Type of place of residence” which is a categorical variable (Urban and Rural) was converted into a dummy variable in SPSS as the following; If Rural = 0, then Urban = 1 and If Urban = 0, then Rural = 1 and the sub-group analysis was conducted in multivariate regression analysis specifically binary logistic regression analysis. The multivariate analysis was conducted through a three stage modeling in determining the influencing factors of HIV testing among reproductive women. Socio-demographic variables were included in the first model. In the second model, both socio-demographic and HIV-related knowledge variables were included. The third model involved variables that explored their risky sexual behaviors and the second model to produce adjusted odds ratios.

All findings of the multivariate analysis were reported with Adjusted Odds Rations (aOR) at 95% Confidence Intervals (95% CI). Sample weights were applied to account for sampling and estimate biases and all analysis were conducted through Complex Samples Analysis in SPSS after Complex Samples Analysis Plan was generated using weight, cluster and strata variables in the Gambian Demographic and Health Survey Datasets (DHS). This approach was to adjust for weight, clustering and stratification of the sampling design in order to produce objective national estimates of the population from the sample taking into account the weights for over or under sampling of specific groups [16,28,29,30].

## 3. Results

### 3.1. Background Characteristics of the Study Population

A total sample of 11,865 respondents were included in this analysis (Table 1). The prevalence of HIV Testing was 42.1% (95% CI: 40.1–44.2%) among survey respondents (Table 1) even though over 97% of study participants have heard of Human Immunodeficiency Virus, (HIV) 97.6% (95% CI: 97.0–98.0%) and other Sexually Transmitted Infections, (STIs) 97.9% (95% CI: 97.4–98.3%). The majority of the respondents were aged 15–19 years (22.3%) and 63.3% of them were married. Nearly half (45.8%) of the study population were in the high income group and 34.8% in the lower income category. By place of residence, 73.7% of them resided in urban areas whilst 26.3% were in the rural centers in the Gambia. About 42.4% of all respondents had secondary education and over 34% had no formal education at all. Again, over half (50.5%) of the study population were currently employed (Table 2). A total of 70.4% of the respondents knew where to be tested whilst 87.9% of them had no knowledge on HIV test kit and how to use it.

Nearly all the respondents (98%) reported that they have not had any STIs within the last 12 months. Health insurance coverage is very low among the survey respondents as 97.2% of them were not covered by the National health insurance. In addition, 96.4% of reproductive women included in this study were Muslims. About 22% have tested at health centers and 59.2% at other places. Again, 61.4% of the participants received results after testing and 41.3% had sexual intercourse within the last 4 weeks. Over half (58%) had more than one sex partner including the spouse. Those who have had sex and others for gifts and cash within the last 12 months accounted for over 98%, whilst 92% of them reported that they had no genital sores and no discharges (Table 2).

### 3.2. Factors Affecting HIV Testing Uptake

HIV Testing was found to be significantly associated with Age (*p* < 0.00), Marital status (*p* < 0.00), Type of place of Residence (*p* < 0.00), Educational status (*p* < 0.00), Respondent currently working/SES (*p* < 0.00), having had any STIs in the last 12 months (*p* < 0.00) as well as Coverage by health insurance (*p* < 0.00), (Table 2). In addition, knowing where to get an HIV test (*p* < 0.00), knowledge and use of test kits (*p* < 0.00), place where last HIV test was taken (*p* < 0.00), having received results for last test (*p* < 0.00) and recent sexual activity (*p* < 0.00) were all associated with HIV testing uptake among reproductive women (Table 2). Furthermore, relationship with most recent sex partner (*p* < 0.00), number of sex partners (*p* < 0.00), having had genital sores (*p* < 0.00), having had genital discharges (*p* < 0.00) and seeking treatment for last STI infection (*p* < 0.00) were all associated with HIV testing.

Findings from the regression analysis (Table 3) showed that respondents’ age was positively associated with HIV Testing uptake. The odds of HIV testing among reproductive women aged 30–34 years (aOR = 5.10, 95% CI = 4.46–7.86) was high compared to those aged 15–19 years. Those aged 20–24 years and 25–29 years were more likely to test (aOR = 3.10, 95% CI = 2.51–3.83), (aOR = 4.52, 95% CI = 3.61–5.54) than those aged 15–19 years. Marital status was also found to be positively associated with HIV testing in the Gambia. Reproductive women who were living with a partner were more likely (aOR = 8.45, 95% CI = 2.64–38.56) to test for HIV than those who were not in any union. Those who were married were more likely (aOR = 5.90, 95% CI = 4.84–7.02) to test compared to those who were not in any union. The odds of testing for HIV among respondents in the middle and high income categories were higher (aOR = 1.31, 95% CI = 1.02–1.57), (aOR = 1.55, 95% CI = 1.16–2.05), respectively, compared to those in the lower income quantiles.

In addition, reproductive women living in rural areas in the Gambia (aOR = 1.72, 95% CI = 1.29–2.29) were more likely to test for HIV than those in the urban centers. Again, respondents from the Jola/Karoninka, Fula/Tukulur/Lorobo, and Non-Gambian ethnic groups, respectively, all have higher odds (aOR-1.31, 95% CI = 1.07–1.85), (aOR = 1.25, 95% CI = 1.01–1.53) and (aOR = 1.38, 95% CI = 1.09–1.74) of testing for HIV in comparison to those from the Mandinka/Jahanka ethnic groups.

Furthermore, respondent’s level of education was found to predict the uptake of HIV testing. The odds of HIV testing was found to be high among reproductive women who had at least primary education (aOR = 1.44, 95% CI = 1.30–1.85), secondary education (aOR = 1.53, 95% CI = 1.36–1.80) and higher education (aOR = 2.75, 95%CI = 1.97–3.57) compared to those with no education at all. Additionally, respondents currently working have a higher likelihood (aOR = 1.15, 95% CI = 1.00–1.31) of testing for HIV as well as those with a previous history of an STI in the last 12 months (aOR = 2.22, 95% CI = 1.44–3.41) compared to those who were not working and those without any history of STIs in the last 12 months.

Reproductive women who knew where to get an HIV test have higher odds of testing (aOR = 2.47, 95% CI = 2.29–7.97) compared to those who do not. Similarly, women who received results of a previous test were more likely (aOR = 5.04, 95% CI = 1.83–6.38) to test than those who did not. Those who were sexually active within the last four weeks were likely to test (aOR = 7.11, 95% CI = 6.13–8.23). In addition, women with history of genital sores were more likely to test (aOR = 2.70, 95% CI = 1.24–5.74). Additionally, those in rural areas with a positive history of genital sore have an increased odds of HIV testing (aOR = 2.85, 95% CI =1.84–3.20). There are, however, reduced odds of testing for those in the urban centers (aOR = 0.58, 95% CI = 0.41–0.81). The odds of HIV testing across rural-urban populations for women who have had sex in the last 12 months for gifts or cash was similarly high, rural (aOR = 7.52, 95% CI = 1.26–46.73), and urban (aOR = 7.68, 95% CI = 6.83–8.64), respectively. Whilst women with a history of genital discharge in the last 12 months within rural areas have increased odds of testing for HIV (aOR = 4.07, 95% CI = 1.33–12.42), there is however lower odds of testing among those in the urban centers (aOR = 0.65, 95% CI = 0.52–0.82).

Reproductive women aged 30–34 years in rural communities were (aOR = 4.02, 95% CI = 3.67–6.17) more likely to test for HIV as well as those in the urban centers (aOR = 5.21, 95% CI = 4.69–9.89). The odds of HIV testing among married women in rural areas was high (aOR = 8.41, 95% CI = 6.56–13.51) as well as those in the urban communities (aOR = 5.40, 95% CI = 4.36–6.66).

Again, HIV testing was higher among respondents who are widowed and living in rural areas (aOR = 7.78 95% CI = 4.69–16.15) than those in the urban areas (aOR = 2.40, 95% CI = 2.05–4.00). Respondents in the middle and high income quantiles in urban areas have high odds of testing (aOR = 1.33, 95% CI = 1.06–2.05), (aOR = 1.65, 95% CI = 1.19–3.25) for HIV, respectively, than those in the rural areas. Those from the Serere ethnic group in the rural areas are more likely (aOR = 1.53, 95% CI = 1.32–2.77) to test for HIV compared to those in the urban areas. Similarly, Non-Gambians in the urban centers have higher odds of HIV testing (aOR = 1.55, 95% CI = 1.25–3.76) compared to those in rural areas. Reproductive women with primary education in rural areas have high odds of testing (aOR = 1.45, 95% CI = 1.17–1.81) as well. Similarly, respondents with primary education in urban centers have increased odds of testing (aOR = 1.69, 95% CI = 1.26–2.09). Similarly, those in the rural and urban settings with secondary education (aOR = 1.34, 95% CI = 1.08–1.93), (aOR = 1.63, 95% CI = 1.29–1.97) are more likely to test for HIV respectively compared to those with no education.

Respondents with higher education in the urban settlements (aOR = 2.65, 95% CI = 2.08–3.86) were more likely to test for HIV compared to those in the rural areas. Respondents currently working and dwelling in urban areas were more likely to procure HIV testing (aOR = 1.40, 95% CI = 1.01–1.43) compared to those in rural communities. Reproductive women with a previous history Sexually Transmitted Infections (STIs) in the last 12 months within the urban population has higher odds of HIV testing (aOR = 2.30, 95% CI = 1.43–3.69) compared to their peers in rural areas. Finally, there are high odds (aOR = 1.61, 95% CI = 1.04–2.48) of HIV testing among respondents who were insured and lived in urban areas compared to those in rural areas.

## 4. Discussion

This study examined the factors that are associated with HIV Testing among reproductive women aged 15–49 years in the Gambia using data from the 2019–2020 Gambian Demographic and Health Survey datasets. The prevalence of HIV testing among survey respondents was 42.1% (95% CI = 40.1–44.2%) even though there is near universal (97%) information about HIV/AIDS and other STIs. This is validated by [3] that reported that general awareness about HIV and other STIs among reproductive women was near universal. However, the prevalence of HIV testing reported in Kenya, Malawi, Uganda, Zambia and other parts of Africa is consistently high [4,31]. The observed variation in prevalence of HIV Testing may be occasioned by interplay of socio-cultural beliefs and lifestyle differences across populations and regions [32] as well as variations in the quality and availability of HIV testing facilities [32,33].

The age of respondent was found to be associated with HIV testing in this study. This finding is consistent with results from other studies [4,31,34,35] that reported significant association between the age of respondent and the uptake of HIV testing. Additionally, this study also showed that marital status influences HIV testing uptake. Reproductive women who were married and those living with their partner were more likely to test for HIV than those who were never in any union. Similarly, those who were separated have higher odds of testing for HIV compared to those who were not in any union. These findings are validated by other studies [4,31,36] that indicated that married women have been found to test for HIV more than those unmarried. This could be explained by perceived risk associated with being infected in previous or current relationships as well as the compulsory testing carried out for couples who intended to marry [31]. Another plausible reason for the increased odds of HIV testing among those separated could be as a result of dynamics in the family system, especially in Sub-Saharan Africa (SSA), where the woman becomes a widow due to the death of the spouse through HIV infection or possible separation as a result of the partner being infected with HIV [37]. Additionally, in some parts of SSA, high risk behaviors and practices, where the woman has to be inherited by surviving relations, and ritual cleansing as well as copulation, increase the woman’s exposure to HIV infection and its transmission. These may therefore explain the increased probability to test for HIV. Another important predictor of HIV testing identified in this study was the Wealth Index of respondents. Reproductive women in the middle and higher wealth index quantile have higher odds of testing for HIV compared to those in the low income quantiles. Similar findings have been reported in [7,9,31,38] where women in the middle and high income quantiles reported an increased likelihood of testing for HIV than those in the lower income group. This may be because women in higher socio-economic positions have better educational experience and exposure, coupled with economic privilege to access and procure voluntary testing and counselling services than those in the lower position.

Furthermore, educational level of respondents was found to be positively associated with HIV testing. Women with primary, secondary or higher education have increased odds of testing for HIV than those with no formal education. This is confirmed by other studies [4,9,35,38,39,40] where study participants with a higher level of education were found to have an increased likelihood of testing in comparison to those with no formal education. This may result from the continuous exposure to HIV and STIs transmission and prevention information that is usually more available in formal school systems than in the communities [41] as well as an improvement in HIV knowledge and empowerment of women through the formal education system to utilize health services. Another plausible reason could be that as women become more enlightened, they become aware of the relevance of knowing one’s status and have an enhanced control over the decision to test.

This study also found a significantly positive relationship between the place of residence of the respondent and HIV testing uptake. The odds of testing among reproductive women in rural areas in the Gambia was higher compared to those in urban centers. This finding is at variance with a study conducted in Ethiopia [39] that reported increased odds of HIV testing among the urban population. This may be explained by the constant availability and accessibility of HIV testing services in the urban areas compared to the rural areas in Ethiopia as well as the differences in the interplay of geographical factors between the two research settings. Reproductive women who were currently working were more probable to test for HIV than those who were not gainfully employed. This finding is similar to a finding in a study conducted in Kenya [38] that indicated that there is an increased likelihood of HIV testing among women who are working compared to their counterparts. This may be due to the fact that working class women are financially independent and can procure HIV testing services.

Again, history of STIs in the last 12 months was identified to be positively associated with HIV testing. Specifically, reproductive women who reported a positive history of STI infection have higher odds of testing for HIV compared to those who do not. This finding agrees with findings from a study conducted in Ghana [42] where women who reported previous history of STIs were more likely to test for HIV. Furthermore, this study revealed largely homogeneity in HIV testing across rural-urban population in the Gambia. Respondent’s age was identified to predict HIV testing across rural and urban areas, however some participants in urban areas have higher odds of testing for HIV compared to those in rural areas. Again, findings from this analysis showed that reproductive women in urban centers who were living with a partner have higher odds of testing for HIV than those in the rural settlements.

Furthermore, women with a positive history of STIs and living in the metropolitan areas in the Gambia were more likely to procure HIV testing than those in the rural areas. These findings are validated by previous literature [20,21,22,23,27] that reported that rural residents were less likely to have tested for HIV. Residents in rural areas have been found to be less likely to receive other types of screening such as breast and cervical cancer screening, invariably suggesting that this trend is not unique to only HIV care [43]. Several reasons have been identified to account for this trend among rural dwellers including high stigma, less perceived risk, loss of privacy and confidentiality, low education levels, inexperienced health workforce in the area of HIV medicine and practice, endemic poverty, inadequate health infrastructure coupled with less access to HIV screening services, most importantly in Sub-Saharan Africa [44,45,46,47].

Survey respondents who reported a positive history of genital sore/ulcers/discharges and those who have had sex in the last 12 months in return for gifts/cash were more likely to procure HIV testing. This finding is consistent with findings from [31] that indicated that women who had a history of genital sores or engaged in risky sexual behaviors were more likely to utilize HIV testing. This is because individuals who engage in risky sexual behaviors are always in perpetual fear and apprehension as well as having precarious sero-status. They become unusually worried and suspicious that they may have infected themselves with HIV and this urges them to seek counselling and testing sessions more than those with no risky behaviors [31,48]. In addition, knowing a place to get tested for HIV was found to be associated with actual testing in this analysis. This is corroborated in studies conducted in Ethiopia [49] and Vietnam [50]. This may be due to the fact that those who knew where to get tested were more exposed to adequate information about testing places and its availability and easy accessibility.

### 4.1. Policy Implication of Findings

The health ministry of Gambia should develop targeted and appropriate evidence-based health interventions for women, especially in rural areas with limited education to enhance HIV testing uptake in line with the generally high knowledge about HIV and other STIs in order to reduce the burdens associated with HIV/AIDS. Government and health sector players should develop policy interventions that promote self-testing for HIV and other STIs in the face of existing stigma associated with HIV/AIDS. Again, community awareness programs on HIV/AIDS and other STIs should be organized frequently to educate community members on the relative importance of early testing and treatment in order to reduce the health-related burden associated with HIV infection. Community opinion leaders and members should be involved in the health program development and implementation to create ownership.

Furthermore, adequate resources should be allocated to health service providers, especially those in the rural areas, and their capacities enhanced to initiate testing and treatment of HIV and its related ailments. Women should be empowered economically and girl child education enhanced and encouraged so that they could become independent socio-economically, thereby affording costs associated with testing and treatment. Additional testing centers should be provided to augment existing ones and testing resources should be made free and easily accessible or subsidized to encourage testing uptake.

Furthermore, continuous health education using the various media must be sanctioned to create awareness about the benefits associated with early testing and treatment of HIV infections in addition to the dangers inherent in delayed testing. Community education should be intensified on stigma that is associated with persons living with HIV in order to encourage others to test early for HIV. The formation of Community Youth volunteer groups should be encouraged where HIV ambassadors share their lived experiences to help demystify the fears associated with HIV infection and its transmission. Women who have reported to the health facilities with histories of genital ulcer and discharges should be counselled and tested for HIV by the health service providers as a matter of government policy.

This will ultimately enhance HIV testing prevalence and reduce the transmission of HIV in the Gambia, essentially contributing to the attainment of the Sustainable Development Goal (SGD) three.

### 4.2. Strengths and Limitations of the Study

The large sample size enhanced the statistical power of this study. Weight application and analysis using a complex samples plan accounted for sampling biases and sampling designs, thereby producing unbiased national estimates. The cross–sectional nature of this study limits causality. In addition, the recall biases due to self-reporting of exposure and health behavior outcomes associated with DHS surveys could have influenced the study findings. Another limitation of this study is that the use of a secondary dataset limits the selection and inclusion of other important variables that could have influenced the study findings. The findings should be interpreted against these.

## 5. Conclusions

Findings from this study showed that age, marital status, wealth index, place of residence, educational level, working status and positive previous history of sexually transmitted infection, recent sexual activity, knowing a place to get tested, and having genital sores/ulcer and discharges the were independent predictors of HIV testing among reproductive women in the Gambia. In essence, health policy makers may consider these factors in developing policies aimed at increasing HIV testing uptake among reproductive women with the view to reducing the health burden associated with HIV as well as its mortalities and morbidities in the Gambia. Future studies should examine the determinants of HIV testing among the male population as well as spatial analysis to identify areas with low testing prevalence. Finally, qualitative studies should be conducted to explore the lived experiences and reproductive women’s perspectives on HIV testing in the Gambia.

## Figures and Tables

**Table 1:** Prevalence of Human Immunodeficiency Virus (HIV) Testing among reproductive women aged 15–49 years in the Gambia, (N = 11,865).

Ever Been Tested for Human Immunodeficiency Virus (HIV)	N (%)	Confidence Interval (CI)
No	6865 (57.9)	55–59.9%
Yes	5000 (42.1)	40.1–44.2%
Ever heard of AIDS		
No	290 (2.4)	2.0–3.0%
Yes	11,575 (97.6)	97.0–98.0%
Ever heard of Sexually Transmitted Infections (STIs)		
No	253 (2.1)	1.7–2.6%
Yes	11,612 (97.9)	97.4–98.3%

**Table 2:** Socio-Demographic features and bivariate results of HIV Testing among reproductive women aged 15–49 years in the Gambia, (N = 11,865).

Characteristics	Frequency N (%)	HIV Testing			
		No N (%)	Yes N (%)	COR (95% CI)	*p*-Values
Age					<0.00 **
15–19	2633 (22.3)	2402 (35.0)	231 (4.6)	[1,1]	
20–24	2181 (18.4)	1432 (20.9)	749 (15.0)	5.44 (4.52–6.56)	
25–29	2248 (18.9)	1062 (15.4)	1186 (23.7)	11.62 (9.68–13.96)	
30–34	1619 (13.6)	581 (8.4)	1039 (20.8)	18.64 (15.05–23.09)	
35–39	1438 (12.1)	540 (7.9)	897 (17.9)	17.30 (14.23–21.02)	
40–44	1028 (8.7)	484 (7.1)	544 (10.9)	11.69 (9.36–14.61)	
45–49	718 (6.0)	362 (5.3)	356 (7.1)	10.23 (8.05–13.01)	
Current Marital Status					<0.00 **
Never in union	3704 (31.2)	3258 (47.5)	446 (8.9)	[1,1]	
Married	7501 (63.3)	3326 (48.4)	4175 (83.5)	9.18 (7.81–10.77)	
Living with partner	25 (0.2)	7 (0.1)	18 (0.4)	18.26 (5.47–61.00)	
Widowed	182 (1.5)	99 (1.4)	83 (1.7)	6.16 (4.24–8.95)	
Divorced	416 (3.5)	163 (2.4)	253 (5.0)	11.36 (8.51–15.15)	
No longer living together/separated	37 (0.3)	12 (0.2)	25 (0.5)	15.77 (6.67–37.28)	
Religion					
Islam					<0.32 *
Christianity &Others	11,443 (96.4)	6637 (96.7)	4806 (96.1)	[1,1]	
Wealth Index Combined	422 (3.6)	228 (3.3)	194 (3.9)	1.17 (0.84–1.62)	
Low					<0.62 *
Middle	4133 (34.8)	2428 (35.4)	1705 (34.1)	[1,1]	
High	2292 (19.4)	1329 (19.4)	963 (19.3)	1.03 (0.87–1.21)	
Type of place of Residence	5440 (45.8)	3108 (45.2)	2332 (46.6)	1.06 (0.92–1.23)	
Urban					<0.00 **
Rural	8747 (73.7)	5182 (75.5)	3565 (71.3)	[1,1]	
Ethnicity	3118 (26.3)	1683 (24.5)	1435 (28.7)	1.24 (1.05–1.45)	
Mandinka/Jahanka					<0.09 *
Wollof	3962 (33.4)	2376 (34.6)	1586 (31.7)	[1,1]	
Jola/Karoninka	1487 (12.5)	826 (12.0)	661 (13.2)	1.20 (0.95–1.50)	
Fula/Tukulur/Lorobo	1311 (11.1)	763 (11.1)	548 (11.0)	1.07 (0.88–1.30)	
Serere	2156 (18.2)	1226 (18.0)	929 (18.6)	1.13 (0.95–1.34)	
Sarahule	425 (3.6)	243 (3.5)	182 (3.6)	1.11 (0.81–1.53)	
Creole/Aku Marabout	868 (7.3)	546 (8.0)	322 (6.4)	0.88 (0.65–1.18)	
Manjago	55 (0.5)	30 (0.4)	25 (0.5)	1.27 (0.63–2.58)	
Bambara	143 (1.2)	71 (1.0)	73 (1.5)	1.53 (1.00–2.34)	
Other	147 (1.2)	92 (1.3)	55 (1.1)	0.90 (0.59–1.36)	
Non-Gambian	110 (1.0)	62 (0.9)	48 (1.0)	1.17 (0.58–2.34)	
Highest Educational level	1201 (10.0)	630 (9.2)	571 (11.4)	1.35 (1.11–1.65)	
No education					<0.00 **
Primary	4119 (34.7)	2239 (32.6)	1880 (37.6)	[1,1]	
Secondary	1854 (15.6)	1001 (14.6)	853 (17.1)	1.01 (0.87–1.18)	
Higher	5021 (42.4)	3228 (47.0)	1793 (35.9)	0.66 (0.58–0.74)	
Respondent currently working	871 (7.3)	397 (5.8)	474 (9.5)	1.42 (1.13–1.78)	
No					<0.00 **
Yes					
Knows a place to get HIV Test	5876 (49.5)	3889 (56.7)	1987 (39.7)	[1,1]	
No & Others	5989 (50.5)	2976 (43.3)	3013 (60.3)	1.98 (1.78–2.20)	
Yes					
Knowledge and use of Test Kits					<0.00 **
Unknown	3515 (29.6)	3515 (51.2)	0 (0.0)	[1,1]	
Known	8350 (70.4)	3349 (48.8)	5000 (100)	7.32 (6.47–8.27)	
Place where last HIV Test was taken					
Government Hospitals					<0.00 **
Government health center/clinic/health post	10,433 (87.9)	6046 (88.1)	4418 (88.4)	[1,1]	
Private hospitals and services	1432 (12.1)	819 (11.9)	582 (11.6)	0.97 (0.81–1.15)	
Others					
Received results for last HIV test					<0.00 **
No & Others	1455 (12.3)	0 (0.0)	1455 (29.1)	[1,1]	
Yes	2632 (22.2)	0 (0.0)	2632 (52.6)	1.00 (0.84–1.18)	
Recent sexual activity					
Never had sex	744 (6.3)	0 (0.0)	743 (14.9)	1.00 (0.81–1.22)	
Active in last 4 weeks	7034 (59.2)	6865 (100)	170 (3.4)	5.05 (3.80–6.71)	
Others					
Relationship with most recent partner					<0.00 **
Spouse	7285 (61.4)	6865 (100)	420 (8.4)	[1,1]	
Others	4580 (38.6)	0 (0.0)	4580 (91.6)	6.63 (5.59–7.86)	
Number of sex partners including spouse in last 12 months					
None	3397 (28.6)	3198 (46.6)	199 (4.0)	[1,1]	<0.00 **
One or more	4894 (41.3)	2100 (30.6)	2794 (55.8)	21.43 (17.38–26.43)	
Had sex in last 12 months in return for gifts, cash and others	3574 (30.1)	1566 (22.8)	2008 (40.2)	20.66 (16.71–25.56)	
No					
Yes % Others					<0.00 **
Had any STI in the last 12 months	6570 (55.4)	2815 (41.0)	3756 (75.1)	[1,1]	
No	5295 (44.6)	4049 (59.0)	1245 (24.9)	0.23 (0.20–0.26)	
Yes					
Had genital ulcer/sores in last 12 months					
No					<0.00 **
Yes	4925 (41.5)	3890 (56.7)	1035 (20.7)	[1,1]	
Don’t know	6940 (58.5)	2974 (43.3)	3966 (79.3)	5.01 (4.36–5.73)	
Had genital discharge in last 12 months					
No					<0.33
Yes					
Don’t know	152 (1.3)	96 (1.4)	57 (1.1)	[1,1]	
Sought advice/treatment for last STI Infection	11,713 (98.7)	6768 (98.6)	4944 (98.9)	1.23 (0.80–1.89)	
No					
Yes					<0.00 **
Others	1622 (98.0)	6800 (99.1)	4822 (96.4)	[1,1]	
Covered by Health Insurance	243 (2.0)	64 (0.9)	179 (3.6)	3.93 (2.67–5.77)	
No					
Yes					
	11,223 (94.6)	6591 (96.0)	4632 (92.6)	[1,1]	<0.00 **
	633 (5.3)	268 (3.9)	365 (7.3)	1.94 (1.51–2.48)	
	9 (0.1)	6 (0.1)	3 (0.1)	0.78 (0.13–4.72)	
	10,951 (92.3)	6456 (94.1)	4495 (89.8)	[1,1]	<0.00 **
	903 (7.6)	400 (5.8)	503 (10.1)	1.80 (1.52–2.14)	
	11 (0.1)	8 (0.1)	3 (0.1)	0.42 (0.86–2.13)	
	402 (3.4)	203 (3.0)	199 (4.0)	[1,1]	<0.00 **
	833 (7.0)	318 (4.6)	515 (10.3)	1.64 (1.16–2.31)	
	10,630 (89.6)	6344 (92.4)	4286 (85.7)	0.68 (0.52–0.90)	
	11,532 (97.2)	6724 (98.0)	4808 (96.1)	[1,1]	<0.00 **
	333 (2.8)	140 (2.0)	193 (3.9)	1.92 (1.43–2.56)	

** Significant at *p* < 0.05; [1,1] = Reference group. * Not Significant at *p* < 0.05.

**Table 3:** Logistic regression analysis of the factors associated with HIV Testing among reproductive women aged 15–49 years in the Gambia, (N = 11,865).

Characteristics	aOR	CI	Rural = 3118		Urban = 8747	
			aOR	CI	aOR	CI
Age						
15–19	[1,1]	[1,1]	[1,1]	[1,1]	[1,1]	[1,1]
20–24	3.1	(2.51–3.83)	3.27	(2.25–4.76)	3.02	(2.61–4.08)
25–29	4.52	(3.61–5.54)	4.28	(3.03–6.58)	4.16	(3.45–6.48)
30–34	5.1	(4.76–7.76)	4.02	(3.67–6.17)	5.21	(4.69–9.89)
35–39	5.77	(4.01–7.30)	4.93	(3.59–6.20)	6.46	(4.54–8.97)
40–44	4.09	(3.19–5.25)	3.42	(2.42–5.22)	4.63	(3.14–5.55)
45–49	3.4	(2.08–4.92)	3.2	(2.30–4.51)	3.12	(2.72–4.27)
Current Marital Status						
Never in union	[1,1]	[1,1]	[1,1]	[1,1]	[1,1]	[1,1]
Married	5.9	(4.84–7.02)	8.41	(6.56–13.51)	5.4	(4.36–6.66)
Living with partner	8.45	(2.64–38.56)	4.04	(0.36–29.62)	11.12	(2.33–53.28)
Widowed	3.7	(2.10–4.56)	7.78	(4.69–16.15)	2.4	(2.05–4.00)
Divorced	5.31	(4.38–7.77)	3.62	(2.69–7.02)	6.82	(4.14–7.18)
No longer living together/separated	8.15	(3.59–18.20)	9.52	(1.04–59.31)	7.42	(3.27–20.74)
Religion						
Islam	[1,1]	[1,1]	[1,1]	[1,1]	[1,1]	[1,1]
Christianity &Others	1.01	(0.50–1.68)	0.65	(0.11–4.69)	0.89	(0.57–1.89)
Wealth Index Combined						
Low	[1,1]	[1,1]	[1,1]	[1,1]	[1,1]	[1,1]
Middle	1.31	(1.02–1.57)	1.02	(0.85–1.50)	1.33	(1.06–2.05)
High	1.55	(1.16–2.05)	0.59	(0.54–1.17)	1.65	(1.19–3.25)
Type of place of Residence						
Urban	[1,1]	[1,1]				
Rural	1.72	1.29–2.29				
Ethnicity						
Mandinka/Jahanka	[1,1]	[1,1]	[1,1]	[1,1]	[1,1]	[1,1]
Wollof	1.29	(0.98–1.57)	1.16	(0.72–1.53)	1.35	(0.78–1.69)
Jola/Karoninka	1.31	(1.07–1.85)	3.86	(2.80–5.71)	1.24	(0.82–1.22)
Fula/Tukulur/Lorobo	1.25	(1.01–1.53)	1.41	(0.67–1.85)	1.2	(0.88–1.64)
Serere	1.22	(0.90–1.79)	1.53	(1.32–2.77)	1.32	(0.70–1.67)
Sarahule	0.7	(0.58–1.11)	0.93	(0.35–1.43)	0.72	(0.55–1.08)
Creole/Aku Marabout	1.55	(0.58–4.13)	1.52	(0.08–25.01)	1.45	(0.41–3.03)
Manjago	1.86	(0.93–3.59)	5.24	(0.99–39.59)	1.84	(0.70–3.87)
Bambara	0.87	(0.59–1.49)	1.74	(0.74–2.30)	0.78	(0.07–1.66)
Other	1.43	(0.59–3.43)	7.52	(1.74–42.29)	1.01	(0.54–2.36)
Non-Gambian	1.38	(1.09–1.74)	1.09	(0.60–1.58)	1.55	(1.25–3.76)
Highest Educational level						
No education	[1,1]	[1,1]	[1,1]	[1,1]	[1,1]	[1,1]
Primary	1.44	(1.30–1.85)	1.55	(1.57–1.91)	1.69	(1.26–2.09)
Secondary	1.53	(1.36–1.80)	1.34	(1.08–1.93)	1.63	(1.29–1.97)
Higher	2.75	(1.97–3.57)	1.08	(0.44–2.89)	2.65	(2.08–3.86)
Respondent currently working						
No	[1,1]	[1,1]	[1,1]	[1,1]	[1,1]	[1,1]
Yes	1.15	(1.00–1.31)	1.08	(0.77–1.31)	1.4	(1.01–1.43)
Knows a place to get HIV Test						
No & Others	[1,1]	[1,1]	[1,1]	[1,1]	[1,1]	[1,1]
Yes	2.47	(2.29–7.97)	5.16	(1.83–6.53)	5.93	(1.44–6.52)
Knowledge and use of Test Kits						
Unknown	[1,1]	[1,1]	[1,1]	[1,1]	[1,1]	[1,1]
Known	0.57	(0.14–2.26)	0.16	(0.01–1.62)	0.96	(0.20–4.52)
Place where last HIV Test was taken						
Government Hospitals	[1,1]	[1,1]	[1,1]	[1,1]	[1,1]	[1,1]
Government health center/clinic/health post	2.45	(0.67–8.88)	2.12	(0.51–8.72)	14.07	(1.03–19.90)
Private hospitals and services						
Others	1.04	(0.23–4.63)	0.81	(0.12–5.29)	0.52	(0.08–3.25)
Received results for last HIV test	3.27	(2.15–3.16)	1.75	(1.66–1.84)	4.92	(1.89–5.01)
No & Others						
Yes	[1,1]	[1,1]	[1,1]	[1,1]	[1,1]	[1,1]
Recent sexual activity	5.04	(1.83–6.38)	5.42	(2.49–6.20)	3.19	(2.47–4.36)
Never had sex						
Active in last 4 weeks	[1,1]	[1,1]	[1,1]	[1,1]	[1,1]	[1,1]
Others	7.11	(6.13–8.23)	0.24	(0.01–0.39)	3.19	(2.20–4.82)
Relationship with most recent partner	0.37	(0.04–3.56)	0.21	(0.14–0.36)	0.32	(0.03–3.37)
Spouse						
Others	[1,1]	[1,1]	[1,1]	[1,1]	[1,1]	[1,1]
Number of sex partners including spouse in last 12 months	0.06	(0.00–0.73)	0.57	(0.09–3.61)	0.01	(0.00–0.13)
None						
One or more						
Had sex in last 12 months in return for gifts, cash and others	[1,1]	[1,1]	[1,1]	[1,1]	[1,1]	[1,1]
No	0.11	(0.00–1.60)	0.92	(0.73–1.17)	0.96	(0.00–1.25)
Yes & Others						
Had any STI in the last 12 months						
No	[1,1]	[1,1]	[1,1]	[1,1]	[1,1]	[1,1]
Yes	0.24	(0.16–3.76)	7.52	(1.26–46.73)	7.68	(6.83–8.64)
Had genital ulcer/sores in last 12 months						
No	[1,1]	[1,1]	[1,1]	[1,1]	[1,1]	[1,1]
Yes	2.22	(1.44–3.41)	1.83	(0.78–4.29)	2.3	(1.43–3.69)
Don’t know						
Had genital discharge in last 12 months	[1,1]	[1,1]	[1,1]	[1,1]	[1,1]	[1,1]
No	2.7	(1.24–5.74)	2.85	(1.84–3.20)	0.58	(0.41–0.81)
Yes	4.93	(1.54–5.17)	8.6	(1.66–44.50)	0.93	(0.62–14.06)
Don’t know						
Sought advice/treatment for last STI Infection	[1,1]	[1,1]	[1,1]	[1,1]	[1,1]	[1,1]
No	0.66	(1.99–2.22)	4.07	(1.33–12.42)	0.65	(0.52–0.82)
Yes	2.18	(2.11–2.30)	2.98	(1.35–7.98)	0.8	(0.41–16.01)
Others						
Covered by Health Insurance						
No	[1,1]	[1,1]	[1,1]	[1,1]	[1,1]	[1,1]
Yes	1.28	(0.32–5.13)	1.65	(0.46–5.94)	0.1	(0.08–1.39)
	2.82	(1.25–5.59)	7.98	(1.35–8.30)	6.32	(1.89–7.66)
	[1,1]	[1,1]	[1,1]	[1,1]	[1,1]	[1,1]
	1.49	(0.92–2.22)	0.51	(0.22–1.17)	1.65	(1.05–2.48)

Significant at *p* < 0.05; [1,1] = Reference group.

## Data Availability

The data used in the conduct of this study are available in the Demography and Health Surveys repository (https://dhsprogram.com/data/available-datasets.cfm (accessed on 15 January 2022).

## Abbreviations

HIV	Human Immunodeficiency Virus
STIs	Sexually transmitted Infections
AIDS	Acquired Immunodeficiency Syndrome
DHS	Demographic and Health Surveys
OR	Odds Ratios
CI	Confidence Intervals
SGDs	Sustainable Development Goals
SPSS	Statistical Package for Social Sciences

## References

[1] Paulin H.N., Blevins M., Koethe J.R., Hinton N., Vaz L.M.E., Vergara A.E., Mukolo A., Ndatimana E., Moon T.D., Vermund S.H. (2015). HIV testing service awareness and service uptake among female heads of household in rural Mozambique: Results from a province-wide survey. BMC Public Health.

[2] UNAIDS (2015). Global AIDS Response Progress Reporting 2015.

[3] Asaolu I.O., Gunn J.K., Center K.E., Koss M.P., Iwelunmor J.I., Ehiri J.E. (2016). Predictors of HIV Testing among Youth in Sub-Saharan Africa: A Cross-Sectional Study. PLoS ONE.

[4] Worku M.G., Tesema G.A., Teshale A.B. (2021). Prevalence and associated factors of HIV testing among reproductive-age women in eastern Africa: Multilevel analysis of demographic and health surveys. BMC Public Health.

[5] Peitzmeier S., Mason K., Ceesay N., Diouf D., Drame F., Loum J., Baral S. (2014). A cross-sectional evaluation of the prevalence and associations of HIV among female sex workers in the Gambia. Int. J. STD AIDS.

[6] Global Burden of Disease Collaborative Network (2018). Global Burden of Disease Study 2017 (GBD 2017) Results.

[7] World Bank Data. https://data.worldbank.org/indicator/SH.DYN.AIDS.ZS?locations=GM.

[8] Teklehaimanot H.D., Teklehaimanot A., Yohannes M., Biratu D. (2016). Factors influencing the uptake of voluntary HIV counseling and testing in rural Ethiopia: A cross sectional study. BMC Public Health.

[9] Bekele Y.A., Fekadu G.A. (2020). Factors associated with HIV testing among young females; further analysis of the 2016 Ethiopian demographic and health survey data. PLoS ONE.

[10] Marinda E., Simbayi L., Zuma K., Zungu N., Moyo S., Kondlo L., Jooste S., Nadol P., Igumbor E., Dietrich C. (2020). Towards achieving the 90–90–90 HIV targets: Results from the south African 2017 national HIV survey. BMC Public Health.

[11] Bekker L.-G., Alleyne G., Baral S., Cepeda J., Daskalakis D., Dowdy D., Dybul M., Eholie S., Esom K., Garnett G. (2018). Advancing global health and strengthening the HIV response in the era of the Sustainable Development Goals: The International AIDS Society—Lancet Commission. Lancet.

[12] Rosenberg N.E., Westreich D., Bärnighausen T., Miller W.C., Behets F., Maman S., Newell M.-L., Pettifor A. (2013). Assessing the effect of HIV counselling and testing on HIV acquisition among South African youth. AIDS.

[13] Kurth A.E., Lally M., Choko A.T., Inwani I.W., Fortenberry J.D. (2015). HIV testing and linkage to services for youth. J. Int. AIDS Soc..

[14] Kharsany A.B., Karim Q.A. (2016). HIV Infection and AIDS in Sub-Saharan Africa: Current Status, Challenges and Opportunities. Open AIDS J..

[15] Gambia Bureau of Statistics (GBoS), ICF (2021). The Gambia Demographic and Health Survey 2019–20.

[16] Diress G., Ahmed M., Adane S., Linger M., Alemnew B. (2021). Barriers and Facilitators for HIV Testing Practice among Ethiopian Women Aged 15–24 years: Analysis of the 2016 Ethiopian Demographic and Health Survey. HIV/AIDS-Res. Palliat. Care.

[17] Mason K., Ketende S., Peitzmeier S., Ceesay N., Diouf D., Loum J., Deen D., Drame F., Baral S. (2013). A Cross-Sectional Analysis of Population Demographics, HIV Knowledge and Risk Behaviors, and Prevalence and Associations of HIV among Men Who Have Sex with Men in the Gambia. AIDS Res. Hum. Retrovir..

[18] van der Loeff M.F.S., Sarge-Njie R., Ceesay S., Awasana A.A., Jaye P., Sam O., Jaiteh K.O., Cubitt D., Milligan P., Whittle H.C. (2003). Regional differences in HIV trends in The Gambia. AIDS.

[19] Sesay C., Chien L.-Y. (2012). Analysis of factors associated with failure to return for an HIV-test result in The Gambia. Afr. J. AIDS Res..

[20] Abate B.B., Kassie A.M., Reta M.A., Ice G.H., Haile Z.T. (2020). Residence and young women’s comprehensive HIV knowledge in Ethiopia. BMC Public Health.

[21] Henderson E.R., Subramaniam D.S., Chen J. (2018). Rural-Urban Differences in Human Immunodeficiency Virus Testing Among US Adults: Findings from the Behavioral Risk Factor Surveillance System. Sex Transm Dis.

[22] Ohl M.E., Perencevich E. (2011). Frequency of human immunodeficiency virus (HIV) testing in urban vs. rural areas of the United States: Results from a nationally-representative sample. BMC Public Health.

[23] Trepka M.J., Fennie K., Sheehan D.M., Lutfi K., Maddox L.M., Lieb S. (2014). Late HIV Diagnosis: Differences by Rural/Urban Residence, Florida, 2007–2011. AIDS Patient Care STDs.

[24] USAID (2022). The DHS Program. Demographic and Health Survey Maryland: ICF International. https://dhsprogram.com/data/available-datasets.cfm.

[25] The DHS Program, Demographic Health Surveys. https://www.dhsprogram.com/What-We-Do/Survey-Types/DHS.cfm.

[26] Mo P.K.H., Lau J.T.F., Xin M., Fong V.W.I. (2019). Understanding the barriers and factors to HIV testing intention of women engaging in compensated dating in Hong Kong: The application of the extended Theory of Planned Behavior. PLoS ONE.

[27] Tran L., Tran P., Tran L. (2020). Influence of Rurality on HIV Testing Practices across the United States, 2012–2017. AIDS Behav..

[28] Bell B.A., Onwuegbuzie A.J., Ferron J.M., Jiao Q.G., Hibbard S.T., Kromrey J.D. (2012). Use of Design Effects and Sample Weights in Complex Health Survey Data: A Review of Published Articles Using Data from 3 Commonly Used Adolescent Health Surveys. Am. J. Public Health.

[29] Davis-Kean P.E., Jager J., Maslowsky J. (2015). Answering Developmental Questions Using Secondary Data. Child Dev. Perspect..

[30] Saylor J., Friedmann E., Lee H.J. (2012). Navigating Complex Sample Analysis Using National Survey Data. Nurs. Res..

[31] Erena A.N., Shen G., Lei P. (2019). Factors affecting HIV counselling and testing among Ethiopian women aged 15–49. BMC Infect. Dis..

[32] Mandiwa C., Namondwe B. (2019). Uptake and correlates of HIV testing among men in Malawi: Evidence from a national population–based household survey. BMC Health Serv. Res..

[33] Salima N., Leah E., Stephen L. (2018). HIV testing among women of reproductive age exposed to intimate partner violence in Uganda. Open Public Health J..

[34] Awopegba O.E., Kalu A., Ahinkorah B.O., Seidu A.-A., Ajayi A.I. (2020). Prenatal care coverage and correlates of HIV testing in sub-Saharan Africa: Insight from demographic and health surveys of 16 countries. PLoS ONE.

[35] Qiao S., Zhang Y., Li X., Menon J.A. (2018). Facilitators and barriers for HIV-testing in Zambia: A systematic review of multi-level factors. PLoS ONE.

[36] Desta W.G., Sinishaw M.A., Bizuneh K.D. (2017). Factors Affecting Utilization of Voluntary HIV Counseling and Testing Services among Teachers in Awi Zone, Northwest Ethiopia. AIDS Res. Treat..

[37] Ba D., Ssentongo P., Sznajder K. (2019). Prevalence, behavioral and socioeconomic factors associated with human immunodeficiency virus in Ghana: A population-based cross-sectional study. J. Glob. Health Rep..

[38] Somefun O.D., Wandera S.O., Odimegwu C. (2019). Media Exposure and HIV Testing Among Youth in Sub-Saharan Africa: Evidence from Demographic and Health Surveys (DHS). SAGE Open.

[39] Ejigu Y., Tadesse B. (2018). HIV testing during pregnancy for prevention of mother-to-child transmission of HIV in Ethiopia. PLoS ONE.

[40] Pepito V.C.F., Newton S. (2020). Determinants of HIV testing among Filipino women: Results from the 2013 Philippine National Demographic and Health Survey. PLoS ONE.

[41] Chimoyi L., Tshuma N., Muloongo K., Setswe G., Sarfo B., Nyasulu P.S. (2015). HIV-related knowledge, perceptions, attitudes, and utilisation of HIV counselling and testing: A venue-based intercept commuter population survey in the inner city of Johannesburg, South Africa. Glob. Health Action.

[42] Nutor J.J., Duah H.O., Duodu P.A., Agbadi P., Alhassan R.K., Darkwah E. (2021). Geographical variations and factors associated with recent HIV testing prevalence in Ghana: Spatial mapping and complex survey analyses of the 2014 demographic and health surveys. BMJ Open.

[43] Doescher M.P., Jackson J.E. (2009). Trends in cervical and breast cancer screening practices among women in rural and urban areas of the United States. J. Public Health Manag. Pract..

[44] Weis K.E., Liese A.D., Hussey J., Gibson J.J., Duffus W.A. (2010). Associations of Rural Residence with Timing of HIV Diagnosis and Stage of Disease at Diagnosis, South Carolina 2001–2005. J. Rural Health.

[45] Sutton M., Anthony M.-N., Vila C., McLellan-Lemal E., Weidle P.J. (2010). HIV Testing and HIV/AIDS Treatment Services in Rural Counties in 10 Southern States: Service Provider Perspectives. J. Rural Health.

[46] Crosby R.A., Yarber W.L., DiClemente R.J., Wingood G.M., Meyerson B. (2002). HIV-Associated Histories, Perceptions, and Practices among Low-Income African American Women: Does Rural Residence Matter?. Am. J. Public Health.

[47] Sison N., Yolken A., Poceta J., Mena L., Chan P.A., Barnes A., Smith E., Nunn A. (2013). Healthcare Provider Attitudes, Practices, and Recommendations for Enhancing Routine HIV Testing and Linkage to Care in the Mississippi Delta Region. AIDS Patient Care STDs.

[48] Khamisa N., Mokgobi M. (2018). Risky sexual behaviour and human immunodeficiency virus (HIV) and acquired immune deficiency syndrome (AIDS) among healthcare workers. South. Afr. J. HIV Med..

[49] Agegnehu C.D., Geremew B.M., Sisay M.M., Muchie K.F., Engida Z.T., Gudayu T.W., Weldetsadik D.S., Liyew A.M. (2020). Determinants of comprehensive knowledge of HIV/AIDS among reproductive age (15–49 years) women in Ethiopia: Further analysis of 2016 Ethiopian demographic and health survey. AIDS Res. Ther..

[50] Huy N.V., Lee H.Y., Nam Y.S., Tien N.V., Huong T.T.G., Hoat L.N. (2016). Secular trends in HIV knowledge and attitudes among Vietnamese women based on the Multiple Indicator Cluster Surveys, 2000, 2006, and 2011: What do we know and what should we do to protect them?. Glob. Health Action.

